# A multi-country time and motion study to describe the experience and burden associated with the treatment of Fabry disease with enzyme replacement therapy with agalsidase alfa and agalsidase beta

**DOI:** 10.1186/s13023-025-03707-2

**Published:** 2025-08-11

**Authors:** Ian Keyzor, Ana Maria Martins, Sema Kalkan Uçar, Hiroyuki Yamakawa, Yin-Hsiu Chien, Nur Arslan, Dau-Ming Niu, Leyla Tümer, Laura Baldock, Simon Shohet, Joseph D. Giuliano

**Affiliations:** 1 Amicus Therapeutics Ltd, Marlow, UK; 2 Universidade Federal de São Paulo, São Paulo, Brazil; 3 Ege University Medical Faculty, Izmir, Turkey; 4 Keio University, Tokyo, Japan; 5 Yokohama Municipal Citizen’s Hospital, Yokohama, Japan; 6 National Taiwan University Hospital, Taipei, Taiwan; 7 Dokuz Eylul University Faculty of Medicine, İzmir, Turkey; 8 Taipei Veterans General Hospital, Taipei City, Taiwan; 9 Gazi University Hospital, Ankara, Turkey; 10 OPEN Health, Marlow, UK; 11 Amicus Therapeutics, Inc., Princeton, NJ USA

**Keywords:** Fabry disease, Real-world, Treatment spent time, Out-of-pocket expenses

## Abstract

**Background:**

Fabry disease (FD) is a rare inherited X-linked lysosomal disorder caused by the deficiency or dysfunction of the enzyme α-galactosidase. This leads to a detrimental accumulation of globotriaosylceramide (Gb3) within multiple cell types. Enzyme replacement therapies (ERTs), including agalsidase alfa and agalsidase beta, can diminish Gb3 levels. Published real-world data on the time, cost and burden associated with the administration of ERTs are limited. These evidence gaps were addressed by generating real-world data quantifying the burden of agalsidase alfa and beta infusions for FD treatment.

**Method:**

The study (ClinicalTrials.gov number: NCT04281537) comprised a prospective time-and-motion and a cross-sectional evaluation of self-reported burden and outcomes associated with ERT administration (including work productivity and out-of-pocket costs) from multiple perspectives (healthcare professionals [HCPs], patients, and caregivers). To assess patient/caregiver experience and burden of ERT, the primary objective was to quantify the total time spent by HCPs in the preparation and administration of a single dose of ERT.

**Results:**

Overall, 76 patients and 6 caregivers were included. Of the 76 patients, (Brazil [*n* = 23], Japan [*n* = 4], Taiwan [*n* = 30] and Turkey [*n* = 19]), 41% were female and the mean (standard deviation [SD]) age at diagnosis was 41.1 (17.1) years. Overall, most patients (70%, *n* = 53) had moderate FD and were treated with agalsidase beta (65%, *n* = 48); this was the predominant ERT administered in Brazil (100%, *n* = 23) and Turkey (74%, *n* = 14); most patients in Japan (75%, *n* = 3) and Taiwan (67%, *n* = 20) received agalsidase alfa. The mean (SD) HCP time spent on all ERT activities was 151.9 (62.5) minutes (2.5 [1.0] hours); the mean (SD) time spent on pre- and post-infusion activities was 20.9 (13.4) (0.3 [0.2] hours) and 12.8 (9.6) minutes (0.2 [0.2] hours), respectively. The mean (SD) time spent by patients for all ERT activities was 368.5 (191.5) minutes (6.1 [3.2] hours); 21% (*n* = 16/76) of patients and 50% (*n* = 3/6) of carers took time off work for an ERT episode.

**Conclusions:**

The multi-region findings provide a more complete picture of the burden associated with ERT administration for FD treatment on patients, caregivers, and HCPs. Results may support further cost-effectiveness modelling for novel treatment approaches and inform treatment decisions and patient management.

**Supplementary Information:**

The online version contains supplementary material available at 10.1186/s13023-025-03707-2.

## Introduction

Fabry disease (FD) is a rare inherited lysosomal disorder which is estimated to affect between 1 in 40 000 to 1 in 170 000 individuals globally [1], although this number may be considerably higher [2]. FD is caused by the deficiency or dysfunction of the enzyme α-galactosidase A, leading to an accumulation of globotriaosylceramide (Gb3) within the lysosomes of multiple cell types, resulting in cellular dysfunction and multi-organ disease [3]. Over time, FD can result in irreversible organ damage, premature renal failure, heart disease, cerebrovascular events, and death [4]. As an X-linked disorder, FD tends to affect males more severely than females, however, females can also present with severe disease [5, 6]. FD is associated with various characteristic clinical signs and symptoms, such as neuropathic pain, gastrointestinal complications, headaches, impaired sweating, vertigo, hearing impairment, angiokeratomas, and cornea verticillata [6]. Reduced health-related quality of life (HRQoL) is more prevalent in patients with FD compared with the general population due to the debilitating symptoms and damage to vital organs [7, 8]. Classical FD phenotype, increasing age, more severe disease and pain have been identified as major contributors to reduced HRQoL [8].

Two well-established enzyme replacement therapies (ERT), agalsidase alfa and agalsidase beta, have demonstrated the potential to effectively diminish Gb3 substrate levels across multiple cell types [9–12]. Both have been approved in Europe since 2001 and subsequently in many other countries worldwide (although only agalsidase beta is currently approved in the United States). Their administration in real-world clinical practice is the focus of this study. However, an oral therapy, migalastat, is also now widely authorized for patients with amenable FD mutations [13]. A third ERT, pegunigalsidase alfa, has gained authorisation in several markets in 2023, including the United States and European Union [14]. ERT preparations need to be administered as an intravenous (IV) infusion typically every 2 weeks by the health care professional (HCP) and therefore lifelong ERT is likely to place a considerable burden on patients, their caregivers and healthcare services. Receiving ERT in a hospital or clinic setting requires frequent visits for IV infusions, which could be stressful and inconvenient for many patients. Infusion times for agalsidase alfa and agalsidase beta can take approximately 40 min and 300 min respectively (according to the summary of product characteristics for existing treatments [15, 16]); however, there will be wider activities associated with treatment administration that will contribute additional time burden to healthcare providers, patients and their caregivers. Additionally, hospital-based treatment is typically conducted in specialized FD centers, which could be located far away from the patient’s residence [17, 18]. In some countries ERT can be administered in the patient’s home under certain circumstances and this is generally perceived to be more convenient than hospital-based therapy [17–19]; this can improve treatment compliance [20] and may help to reduce the burden on healthcare services.

There are currently limited data on the time, cost and burden associated with the administration of ERTs within real-world clinical practice. This study aimed to address these evidence gaps by generating robust real-world data to quantify the current burden of ERT infusions for the treatment of FD across 4 countries.

## Methods

### Study design

This was an international, mixed-methodology, non-interventional study involving adult patients with FD undergoing established treatment with ERT and their caregivers and HCPs. The study comprised a prospective time and motion evaluation and a cross-sectional evaluation of self-reported burden and outcomes associated with ERT treatment (including work productivity and out-of-pocket costs) from multiple perspectives (HCPs, patients, and caregivers). The primary objective was to quantify the total time spent by HCPs in the preparation and administration of a single dose of ERT (with agalsidase alfa or agalsidase beta) in patients with FD. The secondary objective was to quantify the total patient and caregiver time, costs (i.e., out-of-pocket expenses) and work-related absence associated with attendances for the administration of a single dose of ERT (with agalsidase alfa or agalsidase beta) in patients with FD.

### Setting

Patients with FD (aged ≥ 18 years) who had received 4 or more doses of ERT (with agalsidase alfa or agalsidase beta) and attended participating hospitals, treatment centers or community healthcare facilities in 4 countries (Brazil, Taiwan, Japan and Turkey) for administration of ERT (as part of their routine treatment) during the data collection period were eligible for inclusion. Patients who did not give consent for study participation and patients for whom ERT preparation and administration took place exclusively in the home setting were excluded. Access to ERT in a homecare setting varies globally. In Brazil, most patients receive ERT at specific infusion centers, with some centers training patients to perform the infusion at home, initially under supervision. In Japan, home infusion is permitted, however, rarely implemented due to logistical issues, with infusions primarily conducted in designated medical institutions [21]. Furthermore, in Japan, in 2023, the manuals for home ERT for lysosomal disease were developed by Yamakawa et al.. (personal citation – Dr. Yamakawa) with the assistance of the Lysosomal Disease Study Group (Okuyama Group) of the Ministry of Health, Labor and Welfare, Japanese Society for Inherited Metabolic Diseases, and Japanese association of home care medicine. As a result, home ERT has been attracting attention in Japan. In Taiwan, home infusions are not permitted. Designated expert hospitals provide the infusion service. In Turkey, home infusions are also not permitted, and the patients receive infusions in a hospital setting with no specialized infusion centers. This study was reviewed and approved by the independent ethics committee for each participating country.

### Patient recruitment and consent

Adult patients with FD who were receiving ERT treatment were identified from local databases and pharmacy records by the care team at each participating center and were assessed for eligibility in accordance with the inclusion criteria. Patients were approached by a member of their care team, provided with study information, and invited to participate. Patients were approached prior to their next scheduled ERT administration visit, either by telephone, post, or in person during a routine hospital visit. Patients who did not wish to complete the study questionnaires were still eligible for the time and motion study, provided consent for the relevant component had been obtained. Patients eligible for multiple observations in the time and motion study gave initial consent for observation of up to 3 episodes of care, which was confirmed verbally by their healthcare professional (HCP) before each subsequent episode. Study recruitment periods were between August 2021 - March 2022 for the Brazil cohort; September 2021 - January 2022 for the Taiwan cohort; August 2020 – March 2021 for the Japan cohort; and November 2020 - January 2022 for the Turkey cohort. The dates for data collection were between August 2021 – May 2022 for the Brazil cohort; September 2021 – March 2022 for the Taiwan cohort; October 2020 – January 2021 for the Japan cohort and December 2020 – November 2021 for the Turkey cohort.

### Healthcare provider recruitment and consent

HCPs involved in the administration of ERT at participating centers were identified. HCPs were asked to provide consent to be observed during their provision of care and consent from HCPs was verbally confirmed by the observer before each episode of care.

### Caregiver recruitment and consent

Individuals aged 18 years or over who self-identified as caregivers of patients with FD were included in the study. Caregivers (of patients who had already agreed to participate in the study) were approached when attending the ERT administration visit and invited to complete additional questionnaires about their own caregiving experiences; those who were interested provided their informed consent to take part.

### Data collection for time and motion study

**Fig. 1:**
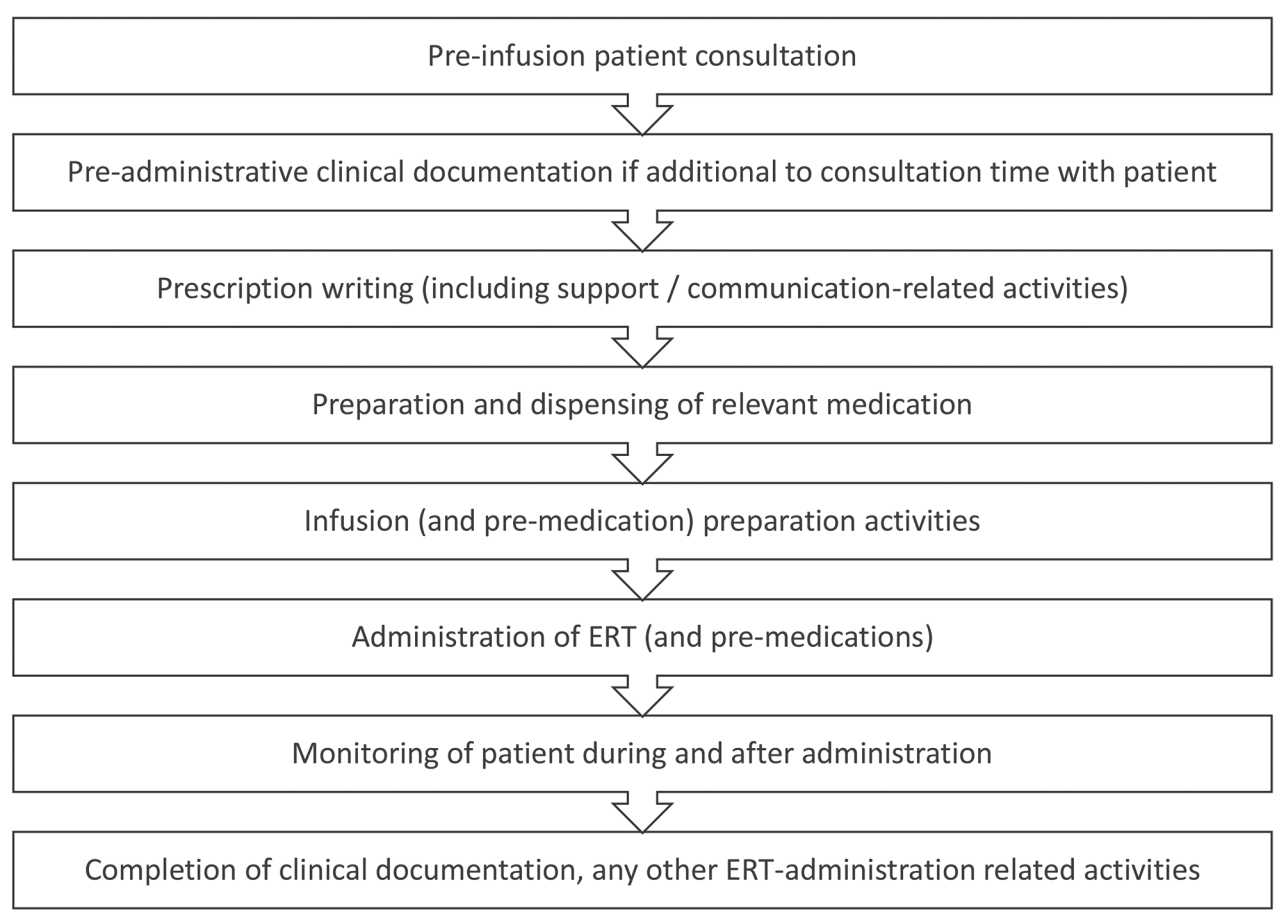
HCP activities timeline. A flow diagram outlining activities conducted by HCPs is presented

Prospective data regarding HCP time associated with the preparation and administration of a single dose of ERT were collected. Data were captured through direct observation of HCPs during the episode of care by an independent external observer or a member of the patient’s care team and an anonymized-coded standardized paper-based case report form (CRF) was used for data collection. The anonymized-coded data from the CRF was transcribed into an electronic data capture (EDC) system through electronic CRFs (eCRFs). Data were collected for each procedure/activity involved in the preparation and administration of a single dose of ERT with agalsidase alfa or agalsidase beta, including the job roles of HCPs involved and the start and stop time for each activity. The tasks undertaken by the HCPs are shown in Fig. 1.

### Patient- and caregiver-reported questionnaires to assess burden and outcomes associated with ERT

**Fig. 2:**
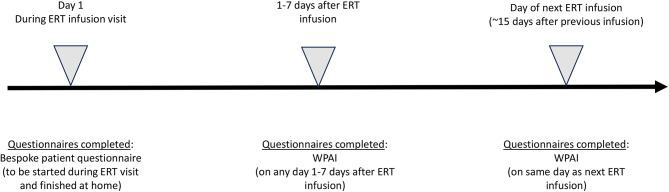
Patient and caregiver questionnaire completion schedule. A summary of all questionnaires completed at each time point

Patients with FD and caregivers were asked to complete a series of questionnaires assessing self-reported burden and outcomes associated with ERT administration (the timeline for questionnaire completion is shown in Fig. 2). On the day of their ERT infusion, patients or caregivers were administered with a series of bespoke questionnaires to evaluate the total time associated with attending the hospital or treatment center for their ERT administration visit (including travel from home), costs (i.e., out-of-pocket expenses), and the time they had spent away from work. Patients or caregivers were asked to finish the questionnaire once they had returned home, so that the total time and costs associated with their visit were captured. The validated Work Productivity and Activity Index: General Health [WPAI: GH] V2.0 questionnaire (WPAI for caregivers [WPAI: CG]) was also administered to measure disease-related impairment in paid work and activities during the past seven days. Patients or caregivers were asked to complete the WPAI twice: once during the 1–7 day period after the patients had received a dose of ERT (ideally within 2 days), in order to capture a seven day period that included an ERT administration visit and secondly on the day the patient or caregiver next attended the hospital or treatment center to receive their next dose of ERT (thereby capturing a seven day period that did not include ERT administration). Participants completed the questionnaires in their native language.

### Data analysis

Analyses were carried out using R statistical software, Stata™ (StataCorp LLC) version 14 and Microsoft Excel™. Data were analyzed separately for each participating country. This study was descriptive in nature; there was no a priori hypothesis to be tested and therefore no direct comparisons between patient subgroups, countries, or treatments were made. Distributions and descriptive statistics of central tendency (medians and arithmetic or geometric means) and dispersion (SD, interquartile range [IQR], range) were presented for quantitative variables. Categorical variables were described with frequencies and percentages; where appropriate, distributions, modes, medians, IQR and range were reported. Where appropriate, 95% confidence intervals (95% CI) were also presented for means and estimates of proportions. Ordinal variables were evaluated using either frequencies and percentages or medians and IQR or both depending on the number of possible values for the variable. All percentages were reported to the nearest whole number and therefore may not add up to 100% due to rounding. Estimates of total time taken to perform ERT-related activities (to address the primary objective) were calculated on a patient level (i.e., calculated as the mean of the mean, whereby the average time spent across all episodes [maximum of 3 per patient] was calculated for each patient, and then the mean total time per patient estimated across all patients based on these estimates). The number of HCP interactions for each country may be more than the total number of patients in that cohort, as on some occasions, multiple HCPs were observed to have spent time on an activity for a single patient: each time a (new) HCP was involved in the activity, their individual start time and stop time was recorded with their job role. Each separate HCP involvement in an activity is referred to as an HCP interaction. Analyses relating to the number of HCP interactions associated with different activities were calculated for the first episode for each patient observed in the study. The WPAI questionnaire was analyzed in accordance with the scoring manual [22], by calculating four percentage impairment scores (higher numbers = greater impairment / reduced productivity) from six items measuring health-related impairment in paid and unpaid work during the past 7 days as follows: (1) absenteeism = percent work time missed, (2) presenteeism = impairment while working / reduced on the job effectiveness, (3) work productivity loss = overall work impairment / absenteeism plus presenteeism, (4) activity impairment = percent impairment in daily activities. Only those who were employed and provided responses for the relevant items were included in the analysis. Severity of FD (mild / moderate / severe) was assigned by the treating clinician based on their clinical judgment according to the following baseline information in the medical records: type of FD (classical / non classical), the number of organs involved, estimated glomerular filtration rate, urine protein levels, left ventricular mass index (LVMi) and white blood cell (WBC) alpha-galactosidase.

## Results

### Participant demographics

Patient demographics are summarized in Table 1. A total of 76 patients with FD were recruited from four countries: Brazil (23 participants), Taiwan (30 participants), Japan (4 participants) and Turkey (19 participants); 6 caregivers were recruited to the study (5 from Taiwan and 1 from Turkey). The mean (SD) age of patients with FD at diagnosis was 41.1 (17.1) years for the overall cohort and 41% of patients were female (Brazil: 30.1 [13.3] years, 57% female; Taiwan: 49.8 [14.4] years, 33% female; Japan: 48.6 [10.3] years, 25% female; and Turkey: 39.0 [18.8] years, 37% female). The mean (SD) age at data collection was 48.9 (15.2) years for the overall cohort (Brazil: 39.2 [12.0] years; Taiwan: 57.0 (13.0) years; Japan: 57.5 [9.5] years; and Turkey: 45.9 [15.5] years.

**Table 1:** Patient demographics

Demographic	Overall	Brazil	Taiwan	Japan	Turkey
Number of participants by country	76	23	30	4	19
Sex *n*, (%)					
Male	45 (59%)	10 (43%)	20 (67%)	3 (75%)	12 (63%)
Female	31 (41%)	13 (57%)	10 (33%)	1 (25%)	7 (37%)
Age at data collection (years), mean (SD)	48.9 (15.2)	39.2 (12.0)	57.0 (13.0)	57.5 (9.5)	45.9 (15.5)
Age at first infusion (years), mean (SD)	43.0 (16.4)	32.5 (12.5)	50.5 (14.5)	53.1 (8.7)	41.9 (17.6)
Age at diagnosis (years), mean (SD)	41.1 (17.1)	30.1 (13.3)	49.8 (14.4)	48.6 (10.3)	39.0 (18.8)
Age at onset of first symptoms (years), mean (SD)	34.0 (22.1)	16.7 (10.3)	47.6 (19.8)	40.3 (24.0)	31.9 (21.5)
*Missing*	*2*	*0*	*0*	*0*	*2*
Employment status *n*, (%*)					
Employed full-time	30 (39%)	8 (35%)	15 (50%)	2 (50%)	5 (26%)
Employed part-time	5 (7%)	2 (9%)	3 (10%)	0 (0%)	0 (0%)
Employed but currently on long term sick leave	2 (3%)	0 (0%)	1 (3%)	0 (0%)	1 (5%)
Unemployed	7 (9%)	2 (9%)	0 (0%)	2 (50%)	3 (16%)
Retired	15 (20%)	2 (9%)	7 (23%)	0 (0%)	6 (32%)
Student	3 (4%)	1 (4%)	0 (0%)	0 (0%)	2 (11%)
Homemaker	7 (9%)	3 (13%)	2 (7%)	0 (0%)	2 (11%)
Other	6 (8%)	4 (17%)	2 (7%)	0 (0%)	0 (0%)
*Missing*	*1*	*1*	*0*	*0*	*0*

*1 patient’s employment status from the Brazil cohort was not documented

Abbreviations: n= total number of patients included in analysis; SD= standard deviation;

### Baseline clinical characteristics

Patient baseline clinical characteristics are summarized in Table 2 and Table S1. In the overall cohort, 16% (*n* = 12) of patients were documented in medical records to have mild FD, 70% (*n* = 53) had moderate FD and 14% (*n* = 11) had severe FD. In total, 56 different FD mutations were documented for the overall sample (Brazil = 21; Taiwan = 23; Japan = 2; Turkey = 10), with the most common mutation being C.639 + 919 G > A in 8 patients. The most commonly documented FD phenotypes in the overall sample were neuropathic pain (64% [*n* = 49]), hypertrophic cardiomyopathy/ left ventricular mass index above normal range (54% [*n* = 41]) and plasmo-Iyso-Gb3 levels above normal (50% [*n* = 38], Table S1). Of the 76 patients taking part in the study, 37% (*n* = 28) were administered with agalsidase alfa and 63% (*n* = 48) were administered with agalsidase beta. The distribution of ERT products administered varied by country sites: in Brazil and Turkey agalsidase beta was the predominant type of ERT administered (100% [*n* = 23] and 74% [*n* = 14], respectively) whereas in Japan and Taiwan most patients (75% [*n* = 3] and 67% [*n* = 20], respectively) received agalsidase alfa.

**Table 2:** Baseline clinical characteristics

Clinical characteristic	Overall	Brazil	Taiwan	Japan	Turkey
Severity of Fabry disease at baseline, *n*	76	23	30	4	19
*n* (%)^*^					
Mild	12 (16%)	7 (30%)	2 (7%)	0 (0%)	3 (16%)
Moderate	53 (70%)	14 (61%)	24 (80%)	4 (100%)	11 (58%)
Severe	11 (14%)	2 (9%)	4 (13%)	0 (0%)	5 (26%)
eGFR at enrolment (closest measurement prior and preferably within 12 months) (mL/min/1.73 m^2^), *n*	66	19	30	3	14
Mean (SD)**	76.6 (31.7)	67.7 (40.9)	77.4 (29.8)	47.8 (33.2)	52.1 (48.1)
*Missing*	*10*	*4*	*0*	*1*	*5*
ERT product administered to patients, *n*	76	23	30	4	19
*n* (%)					
Agalsidase alfa/ biosimilar	28 (37%)	0 (0%)	20 (67%)	3 (75%)	5 (26%)
Agalsidase beta/biosimilar	48 (63%)	23 (100%)	10 (33%)	1 (25%)	14 (74%)
Episode setting where the patient was administered the ERT					
Hospital/infusion setting *n*, (%)	76 (100%)	23 (100%)	30 (100%)	4 (100%)	19 (100%)
Number of infusions received					
1	1	1	0	0	0
2	72	22	30	1	19
3	3	0	0	3	0

*Severity of FD (mild / moderate / severe) determined using baseline information in medical records which included the type of FD (classical / non classical), the number of organs involved, eGFR at enrolment, urine protein levels at enrolment, LVMi at enrolment and WBC alfa-galactosidase at enrolment

** Analysis assumptions were applied: eGFR levels documented as “>90” in medical records were inputted as 90 and “>60” inputted as 60 for the purpose of the analysis

Abbreviations: eGFR: estimated glomerular filtration rate; ERT: enzyme replacement therapy; n: total number of patients included in analysis; SD: standard deviation

### HCP activities associated with ERT treatment

**Fig. 3:**
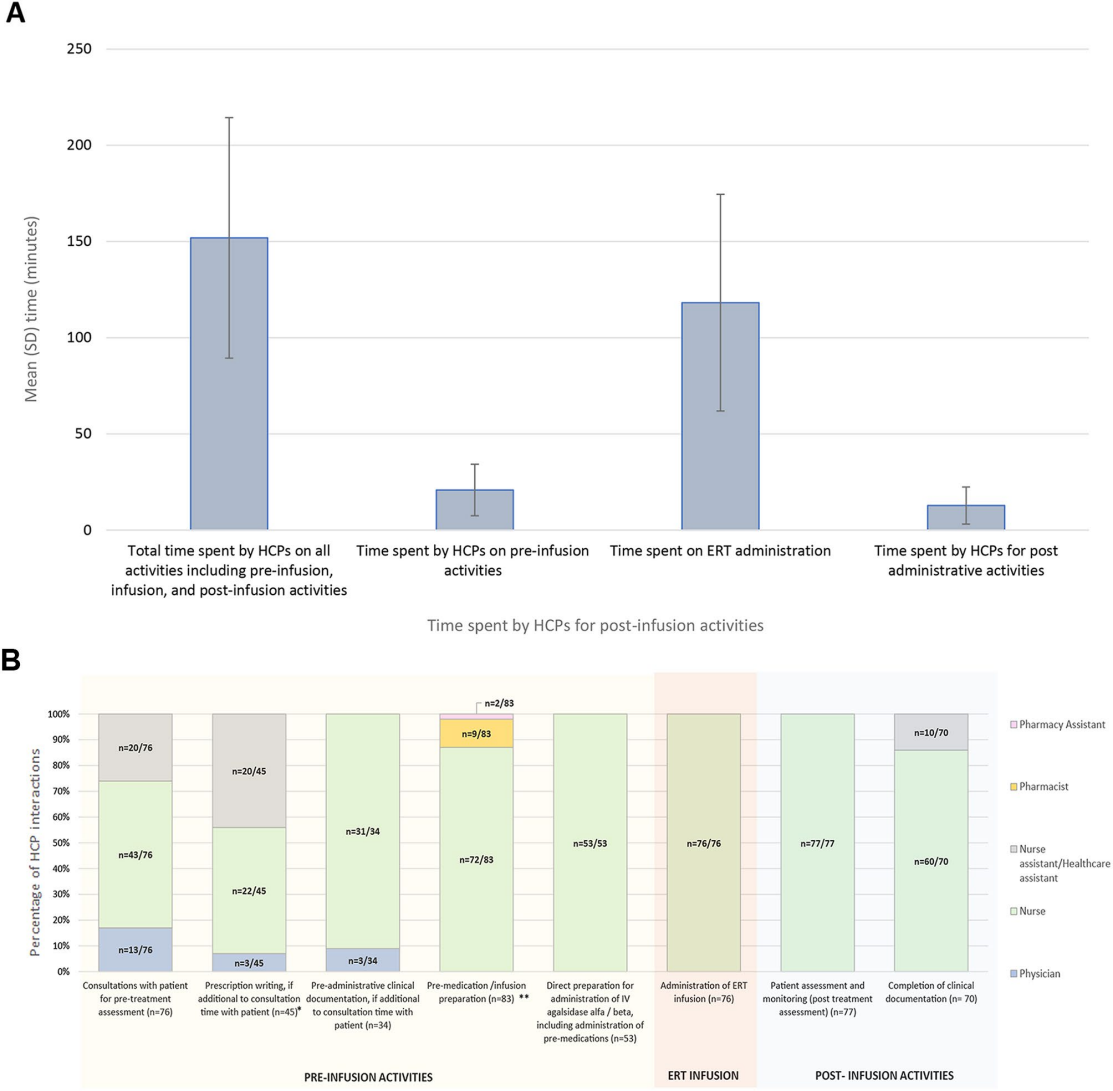
HCP activities associated with ERT treatment. **(A)** The mean standard deviation (SD) time (minutes) spent by HCPs on ERT-associated activities; error bars represent SD. **(B)** Job role of HCPs involved in each ERT-associated activity, expressed as a percentage of all HCP interactions involved in that activity (assessed for the first episode only across all patients; note: the same HCP could have been involved in multiple activities). *Prescription writing includes support-related activities. **‘Pre-medication preparation’ and ‘infusion preparation’ activities include the following: pre-dispensing prescription review, label generation, collation of items prior to assembly, assembly of item/drug reconstitution, labelling of containers, accuracy check, prescription delivery

HCP activities (time) are summarized in Fig. 3A and in Table S2. In the overall cohort, the mean (SD) total time spent by HCPs on all activities including pre-infusion, ERT infusion and post-infusion activities was 151.9 (62.5) minutes. When stratified by country of administration, the mean (SD) total time spent by HCPs on all activities including pre-infusion, infusion, and post-infusion activities was 146.5 (64.4) minutes in Brazil, 147.8 (54.4) minutes in Taiwan, 138.8 (58.2) minutes in Japan and 167.8 (74.2) minutes in Turkey. When separated by activity type the mean (SD) time spent by HCPs on pre-infusion activities in the overall cohort was 20.9 (13.4) minutes. The mean (SD) time spent on ERT administration was 118.2 (56.3) minutes and the mean (SD) time spent on post ERT infusion activities was 12.8 (9.6) minutes. The mean time spent on each activity type was similar across the 4 countries. All recorded (*n* = 76) interactions associated with ERT administration activities were carried out by nurses. The HCP interactions associated with various ERT activities such as consultations, prescription writing, pre-administration clinical documentation, infusion preparation activities, and the administration of IV agalsidase alfa or agalsidase beta are shown in Fig. 3B and Table S3. The total time spent on all activities according to treatment type was a mean (SD) of 110.6 (24.9) minutes for agalsidase alfa and a mean (SD) of 176.0 (65.4) for agalsidase beta. ERT activity stratified by ERT treatment type is shown in Table S3.

### Direct and indirect costs associated with ERT treatment administration

Direct and indirect patient costs relating to ERT treatment administration are summarized in Table 3. In the overall cohort, the mean (SD) total time spent by patients for all ERT infusion activities (including the total time from when the patient left home, to when they returned home and based on the reported time spent travelling, the total waiting time, the total time spent on infusion [pre, during and post], and the other recorded activities) was 368.5 (191.5) minutes. The mean (SD) time was 366.9 (135.2) minutes in the Brazil cohort, 307.9 (99.0) minutes in the Taiwan cohort, 347.5 (49.2) minutes in the Japan cohort and 467.4 (308.8) minutes in the Turkey cohort. In the overall cohort, 21% (*n* = 16/76) of patients reported that they had taken time off work to attend their ERT episode. The proportion of patients known to have taken time off work to attend ERT episodes by country was 30% (*n* = 7/23) of patients in Brazil, 17% (*n* = 5/30) of patients in Taiwan, 50% (*n* = 2/4) of patients in Japan, and 11% (*n* = 2/19) of patients in Turkey. In the overall cohort, patients reported spending a mean (SD) of 6.6 (5.4) paid hours and a mean (SD) of 5.3 (5.4) unpaid hours absent from work. This ranged from a mean (SD) of 3.8 (2.5) paid hours and a mean (SD) of 4.0 (2.6) unpaid hours absent from work in the Taiwan cohort to a mean (SD) of 14.5 (13.4) paid hours and a mean (SD) of 12.8 (15.9) unpaid hours absent from work in the Turkey cohort. The mean (SD) out-of-pocket expenses directly associated with ERT administration were reported as follows (date of conversions: 09 November 2023): in Brazil, the total costs incurred were 166.5 (214.8) R$, with a cost of 6.5 R$ per km, equating to a total cost of 33.9 (43.8) United States dollar (USD)$, with a cost of 1.3 USD$ per km; in Taiwan, the total costs incurred were 709.5 (855.9) NT$, with a cost of 25.0 NT$ per km equating to a total cost of 21.9 (26.5) USD$, with a cost of 0.7 USD$ per km; in Japan, the total costs incurred were 8781.0 (15956.6) ¥, with a cost of 320.0 ¥ per km equating to a total cost of 58.1 (105.7) USD$, with a cost of 2.1 USD$ per km; and in Turkey, the total costs incurred were 470.8 (1005.5)₺, with a cost of 6.0₺ per km equating to a total cost of 16.5 (35.2) USD$, with a cost of 0.2 USD$ per km. Data relating to caregiver costs are summarized in Table S4.

**Table 3:** Direct and indirect patient costs associated with ERT treatment, as reported by patients undergoing ERT

	Overall		Brazil		Taiwan		Japan		Turkey	
Total time spent by patient for ERT infusion activities (refers to the total time from when the patient left home, to when they returned home) (minutes), *n*	74		22		29		4		9	
Mean (SD)	368.5 (191.5)		366.9 (135.2)		307.9 (99.0)		347.5 (49.2)		467.4 (308.8)	
*Missing*	*2*		*1*		*1*		*0*		*0*	
Total patient time spent on travelling (minutes), *n*	74		22		29		4		19	
Mean (SD)	174.1 (136.3)		153.2 (86.6)		157.1 (79.5)		106.3 (68.5)		230.6 (227.6)	
*Missing*	*2*		*1*		*1*		*0*		*0*	
The waiting time at hospital before starting ERT treatment (minutes), *n*	76		23		30		4		19	
Mean (SD)	38.1 (23.8)		42.0 (19.1)		34.9 (16.4)		51.4 (49.6)		35.4 (31.4)	
Patient time off work to attend ERT episode, *n* (%)										
*n*	35		10		19		2		4	
Yes - paid	16 (46%)		7 (70%)		5 (26%)		2 (100%)		2 (50%)	
No - unpaid	19 (54%)		3 (30%)		14 (74%)		0 (0%)		2 (50%)	
*Missing*	41		13		11		2		15	
Patient’s paid/ unpaid hours absent from work	Paid	Unpaid	Paid	Unpaid	Paid	Unpaid	Paid	Unpaid	Paid	Unpaid
*n*	16	19	7	3	5	14	2	0	2	2
Mean	6.6	5.3	7.1	6.7	3.8	4.0	4.0	-	14.5	12.8
SD	5.4	5.4	3.1	4.6	2.5	2.6	1.4	-	13.4	15.9
Costs incurred that were directly related to ERT administration, *n*	-		21		29		4		18	
Mean (SD)	-		166.5 (214.8) R$		709.5 (855.9) NT$		8781.0 (15956.6) ¥		470.8 (1005.5)₺	
*Missing*	-		*2*		*1*		*0*		*1*	
Costs incurred that were directly related to ERT administration - USD$ conversion^†^, mean (SD)^*^	-		33.9 (43.8) USD$		21.9 (26.5) USD$		58.1 (105.7) USD$		16.5 (35.2) USD$	
Cost/Km (travel costs)	-		6.5 R$		25.0 NT$		320.0 ¥		6.0₺	
Cost/Km (travel costs) – USD$ conversion^†^	-		1.3 USD$		0.7 USD$		2.1 USD$		0.2 USD$	

Currency, Brazil (in R$); Taiwan (in NT$); Japan (in ¥); Turkey (in ₺)

* As the countries included in the study may have different healthcare cost settings, there may not be a way to compare across countries

† Date of conversions: 09 November 2023

Abbreviations: cost/km: cost per kilometer; ERT: enzyme replacement therapy; n: total number of patients included in analysis; SD: standard deviation

### Work productivity - WPAI

Results relating to patient WPAI are summarized in Table 4. In the overall cohort, 39% (*n* = 30/76) of patients were in full time employment, 7% (*n* = 5/76) were in part time employment and 3% (*n* = 2/76) were employed but were currently on long term sick leave. The mean (SD) percentage of time missed from work due to health (absenteeism score), during the 1–7 days after the ERT infusion (including the day of the initial infusion), was 5.0 (9.5)%, and the mean (SD) percentage work time missed as reported on the day of the next infusion (capturing a 7-day period that did not include an infusion) was 3.7 (7.3)%. The mean (SD) percent impairment while working due to health (presenteeism) reported during the 1–7 days after the initial ERT infusion was 28.6 (29.5)%, while the presenteeism score was 22.6 (24.9)% reported on the day of the next infusion. Furthermore, the mean (SD) percentage of overall work impairment due to health, as reported during the 1–7 days after the ERT infusion, was 28.9 (27.7)%, while the mean (SD) percentage of overall work impairment due to health as reported on the day of the next infusion was 25.7 (26.4)%. Finally, the percentage of activity impairment due to health, as reported 1–7 days after the initial ERT infusion was 30.8 (29.3)%, while the percentage of activity impairment due to health, as reported on the day of the next ERT infusion, was 32.3 (30.4)%. Results relating to caregiver WPAI are summarized in Table S5.

**Table 4:** WPAI scores

	WPAI completed during the period occurring 1–7 days after ERT infusion				WPAI completed on the day of the next infusion			
	Percent work time missed due to health (absenteeism)	Percent impairment while working due to health (presenteeism)	Percent overall work impairment due to health	Percent activity impairment due to health	Percent work time missed due to health (absenteeism)	Percent Impairment while working due to health (presenteeism)	Percent overall work impairment due to health	Percent activity impairment due to health
**Overall cohort, n**	39	44	38	73	37	38	35	66
Mean (SD)	5.0 (9.5)	28.6 (29.5)	28.9 (27.7)	30.8 (29.3)	3.7 (7.3)	22.6 (24.9)	25.7 (26.4)	32.3 (30.4)
Median (IQR)	0.0 (0.0 to 6.5)	20.0 (0.0 to 50.0)	20.0 (0.0 to 49.1)	20.0 (10.0 to 50.0)	0.0 (0.0 to 5.4)	15.0 (0.0 to 30.0)	20.0 (0.0 to 36.7)	20.0 (10.0 to 57.5)
*Missing*	*37*	*32*	*38*	*3*	*39*	*38*	*41*	*10*
**Brazil cohort, n**	12	15	11	21	12	11	10	17
Mean (SD)	6.9 (12.4)	31.3 (33.2)	35.0 (30.5)	34.8 (34.7)	1.9 (3.7)	29.1 (30.5)	32.6 31.2)	41.8 (36.8)
Median (IQR)	0.0 (0.0 to 10.3)	20.0 (0.0 to 60.0)	28.0 (10.0 to 60.0	30.0 (0.0 to 60.0)	0.0 (0.0 to 1.4)	20.0 (5.0 to 45.0)	25.0 (5.0 to 50.5)	50.0 (0.0 to 80.0)
*Missing*	*11*	*8*	*12*	*2*	*11*	*12*	*13*	*6*
**Taiwan cohort, n**	20	21	20	29	18	20	18	28
Mean (SD)	3.6 (6.9)	21.0 (21.4)	23.6 (22.7)	26.9 (22.5)	3.7 (6.9)	19.0 (19.4)	21.7 (21.2)	25.4 (22.7)
Median (IQR)	0.0 (0.0 to 1.8)	20.0 (10.0 to 30.0)	20.0 (7.5 to 30.0)	20.0 (10.0 to 30.0)	0.0 (0.0 to 2.1)	15.0 (7.5 to 22.5)	20.0 (2.5 to 30.0)	20.0 (10.0 to 30.0)
*Missing*	*10*	*9*	*10*	*1*	*12*	*10*	*12*	*2*
**Japan cohort, n**	2	2	2	4	2	2	2	2
Mean (SD)	0.0 (0.0)	0.0 (0.0)	0.0 (0.0)	30.0 (34.6)	0.0 (0.0)	0.0 (0.0)	0.0 (0.0)	0.0 (0.0)
Median (IQR)	0.0 (0.0 to 0.0)	0.0 (0.0 to 0.0)	0.0 (0.0 to 0.0)	30.0 (0.0 to 60.0)	0.0 (0.0 to 0.0)	0.0 (0.0 to 0.0)	0.0 (0.0 to 0.0)	0.0 (0.0 to 0.0)
*Missing*	*2*	*2*	*2*	*0*	*2*	*2*	*2*	*2*
**Turkey cohort, n**	5	6	5	19	5	5	5	19
Mean (SD)	8.1 (12.5)	53.3 (33.9)	48.5 (34.3)	32.6 (32.5)	9.5 (13.4)	32.0 (32.7)	36.2 (34.1)	37.4 (32.6)
Median (IQR)	4.8 (0.0 to 5.9)	60.0 (35.0 to 77.5)	52.4 (30.0 to 79.0)	30.0 (5.0 to 55.0)	5.9 (0.0 to 9.1)	30.0 (0.0 to 60.0)	30.0 (9.1 to 62.4)	30.0 (10.0 to 70.0)
*Missing*	*14*	*13*	*14*	*0*	*14*	*14*	*14*	*0*

*The WPAI asks questions about the respondent’s work life during the previous 7 days. Therefore, patients were asked to complete the questionnaire twice, once during the period occurring 1–7 days after the initial ERT infusion (thus capturing a 7-day period that *included* an ERT infusion) and on the day of their next infusion, thus capturing a 7-day period that *did not* include an ERT infusion

Scores calculated as per the scoring manual for the WPAI [http://www.reillyassociates.net/wpai_scoring.html]. WPAI outcomes are expressed as impairment percentages, with higher numbers indicating greater impairment and less productivity, i.e., worse outcomes

Abbreviations: ERT: enzyme replacement therapy; IQR: interquartile range; n: total number of patients included in analysis; SD: standard deviation; WPAI: Work Productivity and Activity Index

## Discussion

This international, non-interventional research study was the first real-world study to quantify the burden associated with ERT administration for the treatment of FD. The mixed-methodology design helped provide a more complete picture of the impact of ERT from multiple perspectives. By applying a time-and-motion methodology, it was possible to gain insight into the time burden associated with different ERT activities while minimizing the risk of recall bias. In addition, the use of cross-sectional questionnaires provided additional data about the wider burden associated with ERT administration from a patient and caregiver perspective.

The results of this study demonstrated the significant impact on healthcare resource use both from a service-level and patient perspective. Assuming an HCP administers a single ERT infusion for each patient every two weeks, this extrapolates to approximately 3000 min of staff time (2 full days per year) per patient on average for all the countries. The results also indicated that each year patients spent on average over 9000 min (> 6.5 full days) travelling to and from and attending their ERT appointments. Although the exact time may vary between the countries due to differences in local infrastructure, these results highlight the considerable burden on patients that may have wider-reaching impacts on other aspects of the patient’s life and their health and wellbeing.

The work life of patients was also affected by undergoing ERT treatments. In the overall cohort, patients reported spending a mean (SD) of 6.6 (5.4) paid hours and a mean (SD) of 5.3 (5.4) unpaid hours absent from work when attending the hospital for their ERT infusion. Assuming patients require 26 infusions per year, this represents a mean average of patients losing over 150 h of pay, and 130 unpaid hours, every year. The mean estimated out-of-pocket costs varied across patients and countries (e.g., from approximately USD$17 per patient per ERT episode in Turkey / Taiwan to USD$58 per patient per ERT episode in Japan): the results showed that some patients are incurring expenses that may exceed hundreds or even over a thousand US dollars over the course of a year to travel to their hospital every 2 weeks for their treatment. This economic burden was also reflected in a recent Turkish study which highlighted the high out-of-pocket expenses incurred by patients with inborn errors of metabolism generally [23]. Given that infusion times for particular treatments may be dependent on individual-level characteristics including when tolerance is established [15], the impact of treatment on out-of-pocket expenses and productivity may be greater for certain individuals (e.g. those with a younger age at data collection/ shorter treatment duration).

The results from the WPAI provided further insight into the impact of FD and ERT treatment on the work lives of patients with FD. While the WPAI is well-established and has been applied to many disease areas, to our knowledge this is the first time the WPAI-GH or WPAI-CG have been used for FD. The observed rates of impairment for the four domains generally coincided with those reported in some other disease areas (although were slightly reduced in some cases, particularly when considering results for weeks that did not involve an ERT infusion) [24–28]. As observed in other studies, there were numerically higher rates of presenteeism and overall work and activity impairment relative to absenteeism rates, suggesting that while patients may not be directly taking time off work, they are still experiencing other forms of impairment while at work. Presenteeism due to health conditions has previously been linked to a high proportion of the overall cost associated with a range of diseases [29]. Additionally, presenteeism due to employee health conditions can be associated with significant costs to the employer and contributes to a high proportion of the overall cost associated with various diseases, ultimately due to decreased output as a result of reduced productivity [29]. The percentage impairment scores for the 7-day periods that included the day of the ERT infusion appeared to be higher as opposed to the 7-day period that did not include an infusion. Although no statistical comparisons were carried out, results may suggest work-related impairment associated with FD is enhanced by the additional burden of having to attend hospital for an infusion. It is important to note that the mean age at data collection was approximately 50 years, hence, the impact on WPAI and overall employment status may be different in younger patients; for instance, the mean age was numerically lower for Brazil in the current study.

It was also evident that ERT treatment impacted caregivers. For instance, half of caregivers in this study took time-off work to attend ERT episodes with an impact on work productivity. However, the number of caregivers included was low.

While this study was not designed to directly compare countries, it is notable that there was some variability in the time spent on different activities and the staff members involved between the different countries. These results are likely to reflect service-level differences in the organizational structures and processes at the different hospitals in the participating centers. This may be partially due to the COVID-19 procedures in place as the study was being conducted. Additional research is required to ascertain the factors underlying any country- or center-level differences. As mentioned above, if the manual for home ERT for lysosomal disease (personal citation – Dr. Yamakawa) is fully used by physicians and medical staff involved in lysosomal disease in Japan, it is hoped that the burden on patients and families with lysosomal disease will be improved.

Limitations were associated with this study. For instance, the study required patient consent, which may have caused selection bias and resulted in a study sample that may not be representative of the wider population of interest. The centers were selected based on level of interest in taking part in the study and experience in treating patients with FD with ERT. Therefore, these centers may not be representative of wider clinical practice currently being implemented in the countries of study. It is also possible that the Hawthorne effect could be a limitation of time and motion studies, as the HCPs being observed may work harder and perform better due to being observed, which may not reflect their real-life behaviors when not participating in a study [30]. In addition to this, time and motion studies may be subject to variability in the time endpoints as time and motion studies focus on the dynamic processes. While the time and motion methodology helped to overcome some of the potential limitations that would be associated with collecting data from existing medical records by directly observing practice, data were still missing for some patients in some cases (for example, sometimes there were no data / HCP interactions recorded for certain activities during some episodes). This may have been because these activities were simply not routinely carried out on the same day (perhaps due to service constraints) or it may not have been possible for a data collector to observe the activity that day. Furthermore, there are limitations which are inherently associated with the data analysis. As this is a descriptive study, no analyses to control for confounding were carried out. For ethical reasons, time and motion data were not stratified by individual staff member, although the performance of staff may be a factor in any observed variability. Experience levels of staff performing tasks observed in the study may vary between centers and may not reflect the experience level of staff performing the same tasks outside the study centers, and although all data collectors received standardized training and guidance, there may be some potential for measurement bias across different observers. There are also likely to be differences between the participating countries in local clinical protocols, healthcare settings, and care processes, which may introduce variability in the data collected. While it was not routine practice in the countries of study for ERT preparation/administration to be conducted in the home setting except in exceptional circumstances (hence these patients being excluded), this may also impact the generalizability of the findings to wider countries where homecare is more common. It is also important to note that the study took place following the start of the COVID-19 pandemic and that some results may reflect new procedures and adaptations put in place by hospitals to prevent the spread of COVID-19. Similarly, post-pandemic changes in daily life may have resulted in wider changes for patients and HCPs (e.g. reduced travel times, improved access to healthcare institutions, reduced usage of hygiene measures during ERT infusions), which may in turn impact the time associated with ERT infusions in a post-pandemic world in contrast with what was seen during this study. The study sample size (particularly in Japan) of patients and caregivers was also lower than originally anticipated, potentially impacting the generalizability of the results. It is, however, important to acknowledge the rare nature of FD and that these data therefore still represent a relatively sizable proportion of patients with FD in some countries. This study was funded by a pharmaceutical company (Amicus Therapeutics) who market a Fabry Disease treatment.

## Conclusions

Overall, the findings of this study help fill an important evidence gap and provide a more complete picture of the burden associated with standard-of-care ERT for FD. This is fundamental given the changing treatment landscape for FD. The data provided in this study may therefore be used to support further cost-effectiveness modelling for novel treatment approaches and provide a benchmark of current practice to inform treatment decisions and patient management in the individual countries. In addition, the results may help patients and their caregivers better understand the time burden associated with different treatments. Results highlight the requirement for innovation in ERT drug delivery which could reduce patient and carer burden and associated costs. For instance, exploring reduced infusion times, infusion interval and alternative methods and routes of administration. An oral treatment option for patients with FD who are eligible, may be an appropriate alternative, alongside emerging approaches, such as gene therapies. The aforementioned warrant further investigation. Finally, these findings may also be useful for healthcare providers to help them plan for country-specific service changes that will ultimately improve the efficiency of treatment delivery and overall patient experiences.

## Electronic supplementary material

Below is the link to the electronic supplementary material.

Supplementary Material 1

## Data Availability

Data sharing proposals and requests will be reviewed on a case-by-case basis. Requests for data should be addressed to Joseph D. Giuliano at jgiuliano@amicusrx.com. A medical steering committee will review all requests.
