# Supplementary material for: A multi-country time and motion study to describe the experience and burden associated with the treatment of Fabry disease with enzyme replacement therapy with agalsidase alfa and agalsidase beta
Source: Orphanet J Rare Dis. 2025 Aug 11;20:419. doi: 10.1186/s13023-025-03707-2 (PMC12341357; doi:10.1186/s13023-025-03707-2)
Supplement: Supplementary file 1 — Supplementary Material 1 [file 13023_2025_3707_MOESM1_ESM.docx]

**SUPPLEMENTARY TABLES**

Table S1. FD phenotype (organ involvement/complications), including any prior history

| **FD phenotype,**  n (%) of patients* | **Overall** | **Brazil** | **Taiwan** | **Japan** | **Turkey** |
| --- | --- | --- | --- | --- | --- |
|  | **N=76** | **N=23** | **N=30** | **N=4** | **N=19** |
| *Hypohidrosis* | 19 (25) | 5 (22) | 5 (17) | 1 (25) | 8 (42) |
| *Neuropathic Pain (Acroparesthesia/ Acilli Pain Crisis)* | 49 (64) | 23 (100) | 12 (40) | 0 | 14 (74) |
| *Cornea Verticillata* | 25 (33) | 11 (48) | 5 (17) | 0 | 9 (47) |
| *Angiokeratomas* | 19 (25) | 11 (48) | 1 (3) | 0 | 7 (37) |
| *Hearing Impairment (Deafness/ Tinnitus)* | 11 (14) | 3 (13) | 0 | 0 | 8 (42) |
| *Gastrointestinal (GI) Symptoms related to FD* | 20 (26) | 8 (35) | 4 (13) | 1 (25) | 7 (37) |
| *Plasmo-Iyso-Gb3 levels above normal* | 38 (50) | 0 | 29 (97) | 1 (25) | 8 (42) |
| *Low White Blood Cells (WBC)/ Alpha-galactosidase* | 25 (33) | 0 | 15 (50) | 2 (50) | 8 (42) |
| ***Cardiac Events*** |  |  |  |  |  |
| *Rhythm/ Conduction disturbances* | 11 (14) | 1 (4) | 2 (7) | 3 (75) | 5 (26) |
| *Myocardial Infarction (MI)* | 19 (25) | 5 (22) | 5 (17) | 1 (25) | 8 (42) |
| *Congestive Heart Failure (CHF)* | 6 (8) | 0 | 6 (20) | 0 | 0 |
| *Hypertrophic cardiomyopathy/ Left Ventricular Mass index (LVMi) above normal range* | 41 (54) | 6 (26) | 19 (63) | 3 (75) | 13 (68) |
| *Unstable Angina* | 1 (1) | 0 | 0 | 0 | 1 (5) |
| *Major Cardiac medical procedures* | 3 (4) | 0 | 3 (10) | 0 | 0 |
| *Other*  *Hypertension*  *Bilateral atrial dilatation*  *Palpitations*  *Symptoms of chest tightness* | 3 (4)  1 (1)  2 (3)  1 (1) | 0  0  0  0 | 3 (10)  0  2 (7)  1 (3) | 0  0  0  0 | 0  1 (5)  0  0 |
| ***Renal Events*** |  |  |  |  |  |
| *Reduced estimated Glomerular Filtration Rate (eGFR)* | 20 (26) | 1 (4) | 12 (40) | 1 (25) | 6 (32) |
| *Proteinuria/ Albuminuria* | 32 (42) | 9 (39) | 10 (33) | 2 (50) | 11 (58) |
| *Kidney transplant* | 6 (8) | 4 (17) | 1 (3) | 0 | 1 (5) |
| *Dialysis* | 5 (7) | 1 (4) | 1 (3) | 0 | 3 (16) |
| ***Central Nervous System (CNS) involvement*** |  |  |  |  |  |
| *Brain / Magnetic Resonance Imaging (MRI) changes (white matter lesions) and atrophy* | 15 (20) | 4 (17) | 4 (13) | 0 | 7 (37) |
| *Transient Ischaemic Attack (TIA)/ Stroke / Ischaemic changes* | 4 (5) | 0 | 1 (3) | 0 | 3 (16) |
|  |  |  |  |  |  |
| ***Other***  *Fatigue*  *Depression* | 1 (1)  1 (1) | 0  0 | 0  0 | 0  1 (25) | 1 (5)  0 |

Categories are not mutually exclusive

Table S2. HCP activities associated with ERT treatment.

| **Activity** | **Overall**  **[N=76]** | **Brazil**  **[N=23]** | **Taiwan**  **[N=30]** | | **Japan**  **[N=4]** | **Turkey**  **[N=19]** |
| --- | --- | --- | --- | --- | --- | --- |
| The total time spent by HCPs on all activities including pre-infusion, infusion, and post-infusion activities, mean (SD) | 151.9 (62.5) | 146.5 (64.4) | 147.8 (54.4) | | 138.8 (58.2) | 167.8 (74.2) |
| The total time spent by HCPs on pre-infusion activities (in minutes), mean (SD)^*^ | 20.9 (13.4) | 11.9 (3.4) | 29.6 (15.5) | | 30.3 (14.5) | 16.1 (5.9) |
| The total time spent on ERT administration (in minutes), mean (SD) | 118.2 (56.3) | 123.1 (64.1) | 103.6 (31.3) | | 84.0 (49.3) | 142.6 (70.3) |
| The total time spent by HCPs for post administrative activities, mean (SD) | 12.8 (9.6) | 11.5 (2.6) | 14.7 (12.6) | | 24.5 (8.9) | 9.2 (7.5) |
|  |  |  |  | |  |  |
| Number of HCP interactions associated with consultations for pre-treatment assessment, n (%)^** #^ |  |  |  | |  |  |
| Total number of interactions | 76 | 23 | 30 | | 4 | 19 |
| Physician | 13 (17%) | 0 (0%) | 9 (30%) | | 4 (100%) | 0 (0%) |
| Nurse | 43 (57%) | 23 (100%) | 1 (3%) | | 0 (0%) | 19 (100%) |
| Nurse/ Healthcare assistant | 20 (26%) | 0 (0%) | 20 (67%) | | 0 (0%) | 0 (0%) |
| Pharmacist | 0 (0%) | 0 (0%) | 0 (0%) | | 0 (0%) | 0 (0%) |
| Pharmacy Assistant | 0 (0%) | 0 (0%) | 0 (0%) | | 0 (0%) | 0 (0%) |
|  |  |  |  | |  |  |
| Number of HCP interactions associated with prescription writing, n (%)^** #^ |  |  |  | |  |  |
| Total number of interactions | 45 | 0 | 30 | | 3 | 12 |
| Physician | 3 (7%) | 0 (0%) | 0 (0%) | | 3 (100%) | 0 (0%) |
| Nurse | 22 (49%) | 0 (0%) | 10 (33%) | | 0 (0%) | 12 (100%) |
| Nurse / healthcare assistant | 20 (44%) | 0 (0%) | 20 (67%) | | 0 (0%) | 0 (0%) |
| Pharmacist | 0 (0%) | 0 (0%) | 0 (0%) | | 0 (0%) | 0 (0%) |
| Pharmacy Assistant | 0 (0%) | 0 (0%) | 0 (0%) | | 0 (0%) | 0 (0%) |
|  |  |  |  | |  |  |
| Number of HCPs interactions associated with pre-administrative clinical documentation if additional to consultation time with patient, n (%)^** #^ |  |  |  | |  |  |
| Total number of interactions | 34 | 0 | 30 | | 3 | 1 |
| Physician | 3 (9%) | 0 (0%) | 0 (0%) | | 3 (100%) | 0 (0%) |
| Nurse | 31 (91%) | 0 (0%) | 30 (100%) | | 0 (0%) | 1 (100%) |
| Nurse / healthcare assistant | 0 (0%) | 0 (0%) | 0 (0%) | | 0 (0%) | 0 (0%) |
| Pharmacist | 0 (0%) | 0 (0%) | 0 (0%) | | 0 (0%) | 0 (0%) |
| Pharmacy Assistant | 0 (0%) | 0 (0%) | 0 (0%) | | 0 (0%) | 0 (0%) |
|  |  |  |  | |  |  |
| Number of HCP interactions associated with infusion (and pre-medication) preparation, n (%)^**#^ |  |  |  | |  |  |
| Total number of interactions | 83 | 23 | 30 | | 12 | 18 |
| Physician | 0 (0%) | 0 (0%) | 0 (0%) | | 0 (0%) | 0 |
| Nurse | 72 (87%) | 23 (100%) | 30 (100%) | | 1 (8%) | 18 (100%) |
| Nurse assistant / healthcare assistant | 0 (0%) | 0 (0%) | 0 (0%) | | 0 (0%) | 0 |
| Pharmacist | 9 (11%) | 0 (0%) | 0 (0%) | | 9 (75%) | 0 |
| Pharmacy assistant | 2 (2%) | 0 (0%) | 0 (0%) | | 2 (17%) | 0 |
|  |  |  |  | |  |  |
| Number of HCP interactions associated with the administration of IV agalsidase alfa or agalsidase beta (including administration of pre-medications, n (%)** ^#^ |  |  |  | |  |  |
| Total number of interactions | 53 | 3 | 30 | | 1 | 19 |
| Physician | 0 (0%) | 0 (0%) | 0 (0%) | | 0 (0%) | 0 (0%) |
| Nurse | 53 (100%) | 3 (100%) | 30 (100%) | | 1 (100%) | 19 (100%) |
| Nurse assistant / healthcare assistant | 0 (0%) | 0 (0%) | 0 (0%) | | 0 (0%) | 0 (0%) |
| Pharmacist | 0 (0%) | 0 (0%) | 0 (0%) | | 0 (0%) | 0 (0%) |
| Pharmacy assistant | 0 (0%) | 0 (0%) | 0 (0%) | | 0 (0%) | 0 (0%) |
|  |  |  |  | |  |  |
| Number of HCP interactions associated with all ERT administration, n (%)^**#^ |  |  |  | |  |  |
| Total number of interactions | 76 | 23 | 30 | | 4 | 19 |
| Physician | 0 (0%) | 0 (0%) | 0 (0%) | | 0 (0%) | 0 (0%) |
| Nurse | 76 (100%) | 23 (100%) | 30 (100%) | | 4 (100%) | 19 (100%) |
| Nurse assistant/ healthcare assistant | 0 (0%) | 0 (0%) | 0 (0%) | | 0 (0%) | 0 (0%) |
|  |  |  |  | |  |  |
|  |  |  |  | |  |  |
| Number of HCPs interactions associated with patient assessment and monitoring (post treatment assessment), n (%)^**#^ |  |  |  | |  |  |
| Total number of interactions | 77 | 23 | 30 | | 5 | 19 |
| Physician | 0 (0%) | 0 (0%) | 0 (0%) | | 0 (0%) | 0 (0%) |
| Nurse | 77 (100%) | 23 (100%) | 30 (100%) | | 5 (100%) | 19 (100%) |
| Nurse assistant/ healthcare assistant | 0 (0%) | 0 (0%) | 0 (0%) | | 0 (0%) | 0 (0%) |
| Pharmacist | 0 (0%) | 0 (0%) | 0 (0%) | | 0 (0%) | 0 (0%) |
| Pharmacy assistant | 0 (0%) | 0 (0%) | 0 (0%) | | 0 (0%) | 0 (0%) |
|  |  |  |  | |  |  |
|  |  |  |  | |  |  |
| Number of HCP interactions associated with the completion of clinical documentation, n (%)** ^#^ |  |  |  | |  |  |
| Total number of interactions | 70 | 23 | 30 | | 3 | 14 |
| Physician | 0 (0%) | 0 (0%) | 0 (0%) | | 0 (0%) | 0 (0%) |
| Nurse | 60 (86%) | 23 (100%) | 20 (67%) | | 3 (100%) | 14 (100%) |
| Nurse / healthcare assistant | 10 (14%) | 0 (0%) | 10 (33%) | | 0 (0%) | 0 (0%) |
| Pharmacist | 0 (0%) | 0 (0%) | 0 (0%) | | 0 (0%) | 0 (0%) |
| Pharmacy assistant | 0 (0%) | 0 (0%) | 0 (0%) | | 0 (0%) | 0 (0%) |
| ^*^Pre-infusion activities included: 1. Consultation for pre-treatment assessment 2. Prescription writing 3. Pre-administration clinical documentation 4. Infusion (and pre-medication) preparation activities 5. Administration of IV agalsidase alfa or agalsidase beta (including administration of pre-medications).  ^**^One HCP could have been involved in more than 1 activity.  ^#^Assessed for the first episode only across all patients.  Abbreviations: ERT: enzyme replacement therapy; HCP: healthcare professional; IV: intravenous; SD: standard deviation; n: total number of patients included in analysis | | | |  |  |  |

**Table S3. HCP activities associated with ERT treatment by treatment type**

| **Overall cohort** | **Agalsidase Alfa**  **N=28** | **Agalsidase Beta**  **N=48** |
| --- | --- | --- |
| **Total time spent on all activities (in minutes)** |  |  |
| Mean (SD) | 110.6 (24.9) | 176.0 (65.4) |
| Median (IQR) | 102.0 (98.4 to 117.3) | 151.8 (122.5 to 215.3) |
| Range | 74.5 to 187.0 | 102.0 to 397.0 |
| **Total time spent by HCPs on pre-infusion activities (in minutes)** |  |  |
| Mean | 20.1 | 21.3 |
| SD | 7.1 | 16.0 |
| Median | 20.0 | 14.5 |
| IQR | 17.5 to 21.1 | 10.0 to 25.0 |
| Range | 9.0 to 48.3 | 6.0 to 68.5 |
| **Time spent on ERT administration (in minutes)** |  |  |
| Mean | 82.2 | 139.3 |
| SD | 23.1 | 59.4 |
| Median | 75.0 | 122.3 |
| IQR | 72.9 to 84.1 | 97.1 to 151.6 |
| Range | 50.3 to 163.5 | 82.5 to 363.5 |
| **Total time spent by HCPs for post administrative activities (in minutes)** |  |  |
| Mean | 8.3 | 15.5 |
| SD | 6.4 | 10.2 |
| Median | 6.0 | 11.0 |
| IQR | 6.0 to 6.0 | 10.0 to 21.3 |
| Range | 3.5 to 28.0 | 1.0 to 37.5 |

*Abbreviations: ERT: enzyme replacement therapy; HCP: healthcare professional; IQR: interquartile range; n: total number of patients included in analysis; SD: standard deviation*

Table S4. Caregiver time and costs

| **Activity** | **Overall**  **N=6** | | **Taiwan**  **N=5** | | | **Turkey**  **N=1** | |
| --- | --- | --- | --- | --- | --- | --- | --- |
| Caregiver time-off to work to attend ERT episode, n (%) |  | |  | |  | | |
| Yes | 3 (50%) | | 3 (60%) | | 0 (%) | | |
| No | 3 (50%) | | 2 (40%) | | 1 (100%) | | |
| Paid/unpaid hours absent from work by caregiver |  | |  | |  | | |
|  | Paid hours | Unpaid hours | Paid hours | Unpaid hours | Paid hours | | Unpaid hours |
| n | 1 | 2 | 1 | 2 | - | | - |
| Mean | 6.0 | 3.0 | 6.0 | 3.0 | - | | - |
| SD | - | 4.2 | - | 4.2 | - | | - |
| Median | 6.0 | 3.0 | 6.0 | 3.0 | - | | - |
| IQR | 6.0-6.0 | 1.5-4.5 | 6.0-6.0 | 1.5-4.5 | - | | - |
| Range | 6.0-6.0 | 0.0-6.0 | 6.0-6.0 | 0.0-6.0 | - | | - |
| Missing | *3* | | *2* | | *1* | | |
|  | | |  |  |  |  |  |

*Abbreviations: ERT: enzyme replacement therapy; IQR: interquartile range; n: total number of patients included in analysis; SD: standard deviation*

Table S5. Caregiver WPAI:CG scores / responses (as percentage of impairment)

|  | **WPAI:CG completed during the period occurring 1-7 days after ERT infusion [N=6]** | | | | **WPAI:CG completed on the day of the next infusion**  **[N=6]** | | | |
| --- | --- | --- | --- | --- | --- | --- | --- | --- |
|  | **Percent work time missed due to health (absenteeism)**  **[N=4]** | **Percent impairment while working due to health (presenteeism)**  **[N=4]** | **Percent overall work impairment due to health**  **[N=4]** | **Percent activity impairment due to health**  **[N=6]** | **Percent work time missed due to health**  **(absenteeism)**  **[N=4]** | **Percent Impairment while working due to health**  **(presenteeism)**  **[N=4]** | **Percent overall work impairment due to health**  **[N=4]** | **Percent activity impairment due to health**  **[N=6]** |
| Mean | 0.0 | 17.5 | 17.5 | 18.3 | 5.0 | 17.5 | 21.0 | 16.7 |
| SD | 0.0 | 20.6 | 20.6 | 18.3 | 10.0 | 20.6 | 24.3 | 18.6 |
| Median | 0.0 | 15.0 | 15.0 | 20.0 | 0.0 | 15.0 | 20.0 | 15.0 |
| IQR | 0.0-0.0 | 0.0-32.5 | 0.0-32.5 | 5.0-20.0 | 0.0-5.0 | 0.0-32.5 | 0.0-41.0 | 2.5-20.0 |
| Range | 0.0-0.0 | 0.0-40.0 | 0.0-40.0 | 0.0-50.0 | 0.0-20.0 | 0.0-40.0 | 0.0-44.0 | 0.0-50.0 |
| Missing | *2* | *2* | *2* | *0* | *2* | *2* | *2* | *0* |
| Scores calculated as per the scoring manual for the WPAI [http://www.reillyassociates.net/wpai_scoring.html]. WPAI outcomes are expressed as impairment percentages, with higher numbers indicating greater impairment and less productivity, i.e., worse outcomes. | | | | | | | | |

*Abbreviations: IQR: interquartile range; SD: standard deviation; WPAI-CG= Work Productivity and Activity Index (Caregiver version)*
